# Is Prevention of Postoperative Vomiting Surgery Dependent? A Retrospective Cohort Study of Total Knee Arthroplasty

**DOI:** 10.3390/jpm11101018

**Published:** 2021-10-11

**Authors:** Yan-Yuen Poon, Kuo-Chuan Hung, Wen-Yi Chou, Chih-Hsien Wang, Chao-Ting Hung, Jo-Chi Chin, Shao-Chun Wu

**Affiliations:** 1 Department of Anesthesiology, Kaohsiung Chang Gung Memorial Hospital, Chang Gung University College of Medicine, No. 123, Ta-Pei Rd., Niao-Song Dist., Kaohsiung City 833401, Taiwan; elephant423@gmail.com (Y.-Y.P.); cchwang@cgmh.org.tw (C.-H.W.); timtim0070@gmail.com (C.-T.H.); 2 Department of Anesthesiology, Chi Mei Medical Center, No. 901, Zhonghua Rd., Yongkang Dist., Tainan City 710, Taiwan; ed102605@gmail.com; 3 Department of Orthopedic Surgery, Kaohsiung Chang Gung Memorial Hospital, Chang Gung University College of Medicine, No. 123, Ta-Pei Rd., Niao-Song Dist., Kaohsiung City 833, Taiwan; murraychou@yahoo.com.tw; 4 Department of Anesthesiology, Park One International Hospital, Kaohsiung 813322, Taiwan; jochi731@gmail.com

**Keywords:** postoperative vomiting, risk factors, total knee arthroplasty, surgery dependent

## Abstract

The study of postoperative nausea and vomiting (PONV) has been ongoing since the early days of general anesthesia. The search for risk factors of PONV and the development of new agents to treat PONV are the two main strategies to combat the adverse side effects of general anesthesia. Female sex, non-smoking status, a history of PONV/motion sickness, and postoperative opioid use are the four independent risk factors for PONV derived after a series of prospective studies, evidence-based systematic reviews, and meta-analyses. The two frequently asked questions that arise ask whether risk factors apply to different clinical settings and whether prevention measures of PONV can be surgery dependent. We conducted a comprehensive review of 665 patients who underwent primary total knee arthroplasty (TKA) between January and December 2019. As nausea is subjective and its measurement is not standardized, postoperative vomiting (POV) was used as a study endpoint. The exclusion criteria were desflurane anesthesia, spinal anesthesia, anesthesia without bispectral index monitoring, and day surgery. Three well-recognized risk factors, consisting of body weight, sevoflurane consumption, and postoperative opioid consumption, were not considered as independent risk factors of POV, while female sex, preoperative adductor canal block (ACB), and dexamethasone were identified as being so in this study. The risk of POV in the female sex was 2.49 times that in the male sex; however, when dexamethasone was used, this risk was reduced by >40% compared with no antiemetic use, and by >50% when patients received preoperative ACB compared with those without the block. The clinical characteristics of our TKA patients—female dominance, old age, and their fairly constant body weights that were distinct from those of other surgical patients—suggested that age may play a crucial role in determining the relative contributions of the different risk factors of POV. We concluded that risk factors of POV are dependent on clinical settings. Based on these results, it is reasonable to speculate that a surgery-dependent plan for the prevention of POV is feasible for patients in similar clinical settings.

## 1. Introduction

Postoperative nausea and vomiting (PONV) have been bothersome side effects since the early days of general anesthesia [1,2]. Despite a significant decrease in incidences of PONV over these years [3,4,5] and the unceasing development of new drugs [6,7,8,9,10,11,12,13] for it, most patients still consider PONV their main concern rather than postoperative pain [14,15] and are even willing to pay an extra expense to prevent it [16]. PONV is a crucial obstacle in improving patient satisfaction [17].

Early epidemiological studies identified a number of factors associated with PONV, such as ether, cyclopropane, morphine, intraperitoneal surgery [18], and female sex [19]. However, these so-called risk factors were considered to bear only a correlative relationship with PONV and lacked a causative relationship. Since the early 1990s, multivariate logistic regression analyses have been used to delineate risk factors associated with PONV [5,20]. As a result, many of these studies successfully identified independent predictors for PONV, and some studies even further developed scoring systems for PONV prediction [21,22,23]. However, it was often a matter of concern that these results may only be reflecting authors’ opinions, with increasing doubts as to whether they could be generalized in day-to-day clinical practice. Systematic evidence-based reviews [24,25] integrate the best research evidence and synthesize results of multiple primary studies on a specific issue, minimizing the bias associated with the subjectivity of authors’ opinions and the bias associated with a small number of samples. In 2012, Apfel et al. conducted a systematic review and meta-analysis of 22 prospective studies (*n* = 95,145) on PONV [26]. The results confirmed their previously identified risk factors for PONV [21,22], i.e., female sex, history of PONV/motion sickness, non-smoking status, and postoperative opioid use. These four risk factors are generally accepted as indisputable predictors for PONV, and patients with two of these four risk factors are advised to take necessary precautions to avoid PONV [22].

With increasing advancements in modern technology and a better understanding of the pathophysiology of diseases, many previously proposed surgeries have now become possible [27]. Moreover, clinical settings are becoming increasingly varied. However, it is unclear whether these verified risk factors of PONV are applicable in all clinical settings. Two recently published retrospective studies [28,29] suggest that risk factors for postoperative vomiting (POV) are likely to be dependent on the clinical setting, with some risk factors being modifiable. The present study aims to assess the guiding hypothesis that risk factors of POV are dependent on clinical settings in patients undergoing total knee arthroplasty (TKA).

## 2. Materials and Methods

**Figure 1:**
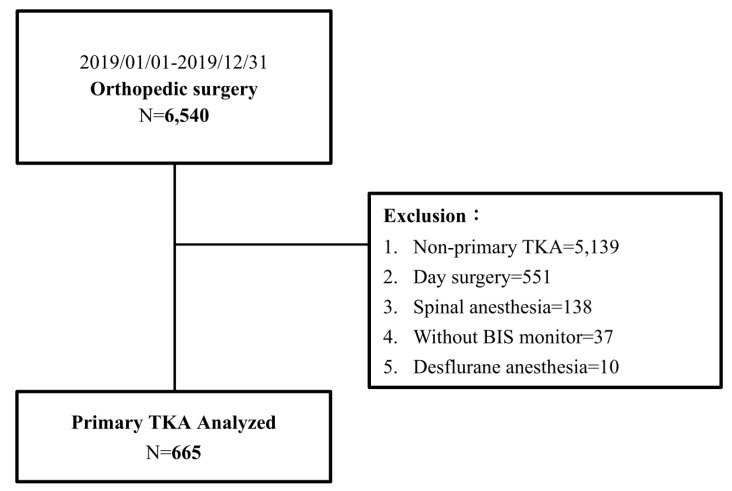
Flow diagram of POV study in patients with primary TKA.

This study was approved by the Institutional Review Board (IRB) of Kaohsiung Chang Gung Memorial Hospital (IRB number: 202100276B0). The requirement for informed consent from patients was waived because their data were de-identified. All methods were performed in accordance with the Declaration of Helsinki and HIPAA Privacy Rule. A total of 6540 orthopedic surgeries were performed between January and December 2019 at our hospital. Data from medical records and anesthesia records were retrieved from the hospital’s electronic database, and data during the stay in the post-anesthesia recovery unit (PACU) and those from routine postoperative daily visits were collected. Patients were visited by well-trained nurse anesthetists within 24 h after surgery. These patients were subjected to the exclusion criteria, which included non-primary TKA, desflurane anesthesia, spinal anesthesia, anesthesia without bispectral index monitoring, and day surgery. Thirteen patients had thirteen missing records of sevoflurane consumption and six missing records of Apfel score, and these missing values were reconstituted with multivariate imputation [30] by *R* with mice [31] package. Finally, 665 patients were included in the analysis (Figure 1).

As nausea is subjective and there is no standard applicable to measure it, we used postoperative vomiting (POV) as an endpoint, which was expressed as a dichotomous unit (vomiter or non-vomiter) in this study. POV was recorded within the first 24 h after surgery. Patients were segregated into two groups for comparison: non-POV and POV groups. Demographic data included sex, age, body weight, ASA physical status (the American Society of Anesthesiologists physical status classification is a five-point scale that assesses a patient’s overall health, from class I to V, with class I being normal and class V being the worst), Apfel score (the higher the score, the higher the chance of postoperative nausea and vomiting from 10% to 79%) [22], and Charlson comorbidity index (a score of zero indicates no comorbidities; the higher the score, the worse the predicated outcome in mortality). The clinical characteristics included duration of anesthesia, sevoflurane consumption, use of antiemetic agents, intraoperative fluid supply, intraoperative urine output, intraoperative use of antihypertensive agents, opioid consumption in the PACU, opioid consumption in the ward, use of patient-controlled analgesia (PCA), and administration of adductor canal block (ACB).

All TKA procedures were performed under sevoflurane general anesthesia, and the anesthesia procedure was compliant with the standard protocol released by our hospital [32]. We deliberately excluded desflurane anesthesia in a limited number of patients. Anesthesia was induced with propofol (1–2 mg/kg), rocuronium (1 mg/kg), or cis-atracurium (0.2 mg/kg). To facilitate endotracheal intubation opioids, fentanyl (1 mcg/kg) or alfentanil (10 mcg/kg) were used, depending on the anesthesiologist’s preference. Sevoflurane (1–1.3 MAC) concentration was titrated against blood pressure and heart rate changes during anesthesia to maintain mean blood pressure and heart rate within 20% of the patient’s resting values, or the BIS score was maintained in the range of 40–60. Fresh gas flow at 1 L/min and 50% oxygen with air was maintained during anesthesia. While intraoperative use of neuromuscular blocking agents or opioids depends on the surgical stimulus, anesthesiologists’ preferences, and objective vital signs, the choice of antiemetic and its use/not use were determined by the known potential risk (e.g., female, history of PONV, etc.) and anesthesiologists’ decisions. Dexamethasone (5 mg) administered at induction and/or ondansetron (8 mg) administered 30 min before the end of surgery were the usual antiemetic prescriptions. The anesthesiologist might not give dexamethasone to diabetic patients for fear of hyperglycemic control.

### Statistical Analysis

Numeric variables were expressed as median (interquartile range, IQR). The Kolmogorov–Smirnov test was used for normality, and normally distributed data were tested using Student’s t-test. The Mann–Whitney U test was used for non-normally distributed data. Categorical variables, expressed as raw numbers (%), were tested using the chi-square test. Reconstitution of missing values was based on Schafer and Schenker methods [30,33] by using R with mice package [31]. Univariate analysis and multiple logistic regression models were used to determine the influence of each variable on POV. Backward stepwise regression was also used. Statistical analyses were performed using SPSS (version 22.0; IBM Corp., Armonk, NY, USA). Statistical significance was set at *p* < 0.05.

## 3. Results

We retrieved 6540 general anesthesia records of patients who underwent orthopedic surgeries between January and December 2019 from our hospital’s electronic database. After excluding surgeries other than primary TKA (*n* = 5139), day surgery (*n* = 551), spinal anesthesia (*n* = 138), anesthesia without BIS monitoring (*n* = 37), and desflurane anesthesia (*n* = 10), 665 patients were finally included in the study (Figure 1). Table 1 summarizes the demographic characteristics of the patients and the distribution of non-POV and POV patients. The demographic data showed that TKA patients were more likely to be elderly and female (73.3%). There was also a significantly larger proportion of female patients in the POV group than in the non-POV group (87.4% vs. 71.2%). The median body weight in the POV group was significantly lower than that in the non-POV group (62.0 kg vs. 68.0 kg). Age, ASA physical status, anesthesia time, Apfel scores, comorbidity index, and the distributions of patients receiving or not receiving ACB were similar between the POV and non-POV groups.

For quantitative statistical analyses, we employed univariate and multiple logistic regression analyses to identify independent risk factors of POV (Table 2). In our univariate analysis, female sex (OR 2.81), body weight (OR 0.97), sevoflurane consumption (OR 55.04), and morphine milligram equivalent consumption at wards (OR 10.83) were each identified as risk factors of POV. In our multivariable logistic regression model, body weight, sevoflurane consumption, and morphine milligram equivalent consumption at wards were not considered risk factors of POV, while female sex (OR 2.49), the use of one antiemetic (OR 0.57), and patients with preoperative ACB block (OR 0.48), were detected as independent risk factors. In our backward stepwise regression model (Table 3), female sex (OR 2.58) and preoperative ACB (OR 0.53) remained independent risk factors of POV, while the use of an antiemetic (OR 0.62) was not detected as an independent risk factor (*p* = 0.054).

## 4. Discussion

With a better understanding of the pathophysiology of PONV [34,35,36,37,38,39], precise administration of antiemetic agents that target various trigger zones of nausea and vomiting is possible [6,7,8,9,10,11,12,13]. In addition, previous studies [21,26,40,41] have identified independent risk factors of PONV, and the incidence of PONV has considerably reduced over the years [3,4,5]. Female sex, a history of PONV/motion sickness, non-smoking status, and postoperative opioid use are four well-known risk factors of PONV that were identified after a series of studies. Female sex remained a strong predictor of POV in our multivariate analysis model. One important finding revealed in this study was that body weight, sevoflurane consumption, and postoperative morphine consumption were factors related to POV in the univariate analysis, but they were not considered independent risk factors of POV in our multivariable logistic regression model. The discrepancy between the univariate and multivariate regression results relies on the fact that multivariate analysis considers interactions of various pro-vomiting factors instead of one factor at a time, as in the univariate analysis. After counterbalancing the effects of various factors, they no longer contributed significantly to the POV under real-world data. Our two recent reports [28,29] suggested that clinical settings could play a crucial role in determining the relative effects of factors on POV in various surgeries.

Another interesting finding was that most patients were older than 65 years, with a median age of 70 years. It is reasonable to speculate that the relative homogeneity of age in this study is particularly important in deciphering the relative roles of factors related to POV. First, previous studies have shown that young patients are prone to developing PONV [21,42,43,44]. Apfel et al. reported that the risk of PONV decreases by 0.88 times per decade increase in age [26]. A recent study of ours [28] showed that the risk of POV in patients aged ≥70 years was 0.29 times that in patients aged 20 years and older in laparoscopic cholecystectomy. Second, previous studies [42,45,46,47,48,49,50] have proved that body weight plays divergent roles in triggering PONV, and another of our recent studies [29] showed that lower body weight favors a higher incidence of POV in trauma surgeries. While bodyweight was not found to be an independent risk factor in this study, the absence of a significant effect of body weight on POV may be attributed to the fact that the body weights of the patients were fairly constant within a reasonable range, and the effect of age in alleviating POV was dominant over bodyweight effect. Third, inhalational anesthetics [51,52,53] and perioperative opioids [54,55,56] are generally considered to trigger PONV. However, sevoflurane and postoperative morphine consumption were not independent risk factors for POV in TKA patients. Previous studies [57,58] have shown the minimum alveolar concentration (MAC) for sevoflurane in elderly patients (mean age 71.4) to be 1.48%, which is lower than that in children and adults. Opioids [59,60] are effective in treating either acute or chronic pain, and previous studies [61,62,63] show that older patients require lower doses of opioids. It has also been reported [64] that older patients who underwent hip or knee arthroplasty under general anesthesia received lower doses of opioids perioperatively. The age effects on MAC and opioid dosage among the patients in this study may explain why sevoflurane and postoperative morphine do not appear to be significant contributors to POV.

A third interesting finding of our study was that proper and adequate analgesic treatment would decrease the odds of developing POV. Patients who received preoperative ACB were 0.48 times less likely to have POV when compared to their counterparts without the block. It is reasonable to speculate that preoperative ACB would further reduce either inhalational anesthetics or intraoperative opioid consumption in elderly patients, and these two agents are well-known triggers for POV. The reduction in inhalational anesthetics and intraoperative opioids in patients receiving preoperative ACB was supported by our recently published study on the timing of performing ACB in TKA [65].

A fourth interesting finding of our study was that the use of one antiemetic (OR 0.57) lowered the risk of POV by >40%, compared with no antiemetic administration. The primary antiemetic administered to the patients was dexamethasone. Dexamethasone is generally considered an effective antiemetic and is devoid of serious side effects [66,67,68]. Our results show that, when an additional antiemetic is used, it offers no further benefit, although a previous study [69] concludes that the use of two antiemetics is better than one. A possible reason for this discrepancy may be that only 36 patients received two types of antiemetics in this study, so more patients are needed for further clarification. One crucial question relating to the use of dexamethasone in this study was whether its use would dilute the pro-POV effects of various triggering factors. First, in our multivariate regression model, female sex remained the strongest triggering factor for POV, followed by preoperative ACB. It is reasonable to speculate that the dexamethasone effect could not be totally excluded in this study; however, it did show that female sex and preoperative ACB are potent factors affecting POV. In our backward stepwise regression model (Table 3), female sex and preoperative ACB remained predictors for POV after the elimination of variables in a stepwise regression approach that started with a full model, in which dexamethasone was one of the variables under discrimination.

The current strategy for the prevention of PONV or POV is based on risk factor identification, and the Apfel risk scoring system recommends that, for patients with at least two of the four well-known risk factors, necessary precautions should be observed to lower the risk of PONV [22]. An often-asked question is whether these measures are “one size fits all.” Our previous studies [28,29] have shown that different clinical settings may have various contributing factors. Speculatively, an important implication of the present study was that the prevention of POV could be surgery dependent. Our study suggested that aged patients undergoing TKA would benefit only from preoperative ACB and an antiemetic.

There are a few limitations to our study. First, our study may have a potential bias of either including or excluding cases with missing data from the statistical analyses. Reconstitution of missing values based on Schafer and Schenker methods and backward stepwise regression was employed in the study to reduce the associated bias as much as possible. Second, postoperative nausea (PON) is occasionally as bad as vomiting; however, an evaluation of its severity or occurrence was not available in this study. Third, PON and PONV are common complications of general anesthesia; nevertheless, they were not included in the analysis. Fourth, the mechanism by which age suppresses POV was not addressed in this study.

## 5. Conclusions

We concluded the following: First, any independent risk factor identified would probably be a consequence of the interactions between various potential risk factors, and old age is likely a potent dominant entity that determines the relative roles of pro-POV factors in TKA, though further elucidation is needed. Second, dexamethasone could provide effective antiemetic effects at a very low cost, irrespective of Apfel risk scores. Third, preoperative ACB could be a better choice for both POV prevention and pain reduction after TKA. Most importantly, further prospective studies are necessary to verify the findings and the feasibility of making a surgery-dependent plan for POV prevention.

## Figures and Tables

**Table 1:** Demographic characteristics of 665 patients with or without postoperative vomiting after primary TKA.

Features	N (%)/Median (IQR) (N = 665)	None-POV (N = 578)	POV (N = 87)	*p* Value
Sex				
Male	178 (26.8%)	167 (28.9%)	11 (12.6%)	0.001
Female	487 (73.2%)	411 (71.1%)	76 (87.4%)	
Age (years)	70 (65–75)	70 (65–75)	71 (65–76)	0.731
Weight (kg)	67 (59–76)	68 (60–77)	62 (55–70)	<0.001
ACB Block				
None	287 (43.2%)	243 (42.0%)	44 (50.6%)	0.167
Pre-operative	226 (34.0%)	204 (35.3%)	22 (25.3%)	
Post-operative	152 (22.9%)	131 (22.7%)	21 (24.1%)	
ASA				
II	408 (61.4%)	351 (60.7%)	57 (65.5%)	0.422
III	257 (38.6%)	227 (39.3%)	30 (34.5%)	
Anesthesia time (h)	3.17 (2.83–3.50)	3.18 (2.85–3.50)	3.07 (2.75–3.45)	0.071
Apfel Score				
0	56 (8.4%)	51 (8.8%)	5 (5.7%)	0.570
1	154 (23.2%)	137 (23.7%)	17 (19.5%)	
2	311 (46.8%)	267 (46.2%)	44 (50.6%)	
≥3	144 (21.7%)	123 (21.3%)	21 (24.1%)	
Charlson Comorbidity Index				
0	173 (26.0%)	152 (26.3%)	21 (24.1%)	0.410
1	282 (42.4%)	247 (42.7%)	35 (40.2%)	
2	138 (20.8%)	121 (20.9%)	17 (19.5%)	
≥3	72 (10.8%)	58 (10.0%)	14 (16.1%)	

**Table 2:** Univariate and multivariate logistic regression of postoperative vomiting risk.

Variables (Unit)	N (%)/	Univariate		Multivariable	
	Median (IQR)	OR (95% CI)	*p*-Value	OR (95% CI)	*p* Value
Sex-Male	178 (26.8%)	1		1	
Sex-Female	487 (73.2%)	2.81 (1.46–5.42)	0.002	2.49 (1.19–5.20)	0.015
Age-	70 (65–75)	1.01 (0.98–1.04)	0.706	1.00 (0.97–1.03)	0.977
ACB Block-None	287 (43.2%)	1		1	
Nerve Block-Pre-op	226 (34.0%)	0.60 (0.35–1.03)	0.062	0.48 (0.26–0.87)	0.016
Nerve Block-Post-op	152 (22.9%)	0.89 (0.51–1.55)	0.671	0.66 (0.36–1.22)	0.188
Weight (kg)	67 (59–76)	0.97 (0.95–0.99)	0.001	0.98 (0.95–1.00)	0.065
ASA II	408 (61.4%)	1		1	
ASA III	257 (38.6%)	0.81 (0.51–1.31)	0.393	0.74 (0.44–1.24)	0.252
Apfel Score 0	56 (8.4%)	1		1	
Apfel Score 1	154 (23.2%)	1.27 (0.44–3.61)	0.659	1.72 (0.56–5.27)	0.344
Apfel Score 2	311 (46.8%)	1.68 (0.64–4.44)	0.295	1.68 (0.58–4.80)	0.337
Apfel Score 3&4	144 (21.7%)	1.74 (0.62–4.87)	0.290	2.19 (0.70–6.85)	0.180
Duration (h)	3.17 (2.83–3.50)	0.69 (0.45–1.05)	0.081	0.70 (0.44–1.11)	0.133
Sevoflurane consumption (mL/h)	0.20 (0.17–0.25)	55.04 (1.50–2019.69)	0.029	2.84 (0.04–201.49)	0.631
Crystalloid (mL/h/kg)	2.51 (2.04–3.28)	0.96 (0.76–1.21)	0.734	0.82 (0.61–1.11)	0.199
Intraoperative Urine (mL/h/kg)	1.00 (0.00–2.02)	0.89 (0.74–1.08)	0.224	0.87 (0.70–1.07)	0.172
PCA-none	616 (92.6%)	1		1	
PCA-Yes	49 (7.4%)	0.57 (0.20–1.63)	0.295	0.73 (0.21–2.57)	0.624
Kinds of anti-emetics-none	296 (44.5%)	1		1	
One	333 (50.1%)	0.66 (0.42–1.05)	0.081	0.57 (0.34–0.94)	0.028
Two	36 (5.4%)	0.48 (0.14–1.64)	0.241	0.40 (0.09–1.75)	0.224
Kinds of anti-hypertension-none	223 (33.5%)	1		1	
One	349 (52.5%)	1.00 (0.61–1.64)	0.996	0.94 (0.55–1.61)	0.823
Two	93 (14.0%)	0.78 (0.36–1.66)	0.511	0.72 (0.32–1.63)	0.424
Intraoperative MME (mg/kg)	0.23 (0.18–0.29)	10.83 (1.51–77.69)	0.018	7.04 (0.73–68.03)	0.092
MME at PACU (mg/kg)	0.02 (0.01–0.02)	0.62 (0.00–1157.62)	0.901	2.03 (0.00–5332.59)	0.860
MME at Ward (mg/kg)	0.10 (0.09–0.11)	0.44 (0.07–2.75)	0.377	0.59 (0.08–4.38)	0.607

**Table 3:** Stepwise multivariate logistic regression of postoperative vomiting risk (backward selection).

Variables (Unit)	N (%)/	Multivariable	
	Median (IQR)	OR (95% CI)	*p* Value
Sex-Male	178 (26.8%)	1	
Sex-Female	487 (73.2%)	2.58(1.29–5.14)	0.007
ACB Block-None	287 (43.2%)	1	
Nerve Block-Pre-op	226 (34.0%)	0.53(0.30–0.93)	0.028
Nerve Block-Post-op	152 (22.9%)	0.71(0.39–1.28)	0.251
Weight (kg)	67 (59–76)	0.98(0.96–1.00)	0.046
Duration (h)	3.17 (2.83–3.50)	0.70(0.45–1.09)	0.110
Intraoperative Urine (mL/h/kg)	1.00 (0.00–2.02)	0.83(0.68–1.01)	0.059
Kinds of anti-emetics-none	296 (44.5%)	1	
One	333 (50.1%)	0.62(0.38–1.01)	0.054
Two	36 (5.4%)	0.38(0.11–1.35)	0.134
Intraoperative MME (mg/kg)	0.23 (0.18–0.29)	6.28(0.89–44.53)	0.066

## Data Availability

The data presented in this study are available from the corresponding authors upon reasonable request.
